# Discrimination of Meat Produced by *Bos taurus* and *Bos indicus* Finished under an Intensive or Extensive System

**DOI:** 10.3390/ani10101737

**Published:** 2020-09-24

**Authors:** Maria C. Bressan, Erika C. Rodrigues, Lizandra V. Rossato, Isabel Neto-Fonseca, Susana P. Alves, Rui J.B. Bessa, Luis T. Gama

**Affiliations:** 1 Faculdade de Medicina Veterinária, Universidade Lusófona, Campo Grande, 376, 1749-024 Lisboa, Portugal; mcbressan1@gmail.com; 2 CIISA, Faculdade de Medicina Veterinária, Universidade de Lisboa, 1300-477 Lisboa, Portugal; isaneto@fmv.ulisboa.pt (I.N.-F.); susanaalves@fmv.ulisboa.pt (S.P.A.); rjbbessa@fmv.ulisboa.pt (R.J.B.B.); 3 Universidade Federal de Lavras, Lavras, Minas Gerais 37200-000, Brazil; rodrigueserikac07@gmail.com (E.C.R.); lizandrarossato@gmail.com (L.V.R.); 4 IFMT, Campus Cuiabá, Bela Vista, Mato Grosso 78050-560, Brazil; 5 Instituto Mineiro de Agropecuária, Belo Horizonte, Minas Gerais 31630-901, Brazil

**Keywords:** cattle, discriminant analyses, fatty acids, meat certification

## Abstract

**Simple Summary:**

This research was carried out to investigate the usefulness and reliability of meat quality traits such as physicochemical properties and fatty acid profiles to discriminate between meat produced by taurine and zebu cattle, finished on pasture or grain. This approach is of utmost importance to assess the authenticity of meat products, particularly when beef is certified based on criteria such as breed and finishing system. Our results using discriminant analyses indicate that meats originating from pasture- or grain-finishing can be discriminated with high reliability, essentially on the basis of their fatty acid profile. The global distinction of meat from the two genetic groups is somewhat more difficult to achieve reliably. Still, prior knowledge of the finishing system used to produce meat from a given genetic group enhances the trustworthiness of the assignment procedure, allowing the reliable allocation of meat samples originating from *Bos indicus* and *Bos taurus* finished on pasture or grain, with fatty acid profiles being better discriminating factors than physicochemical properties. These results can be adopted as tools to be used in meat certification programs.

**Abstract:**

Meat obtained under commercial conditions shows considerable variability, mostly due to genetic background and production system. In this study, meat physicochemical properties and fatty acid profiles were analysed to investigate the feasibility of using them as tools to discriminate between meats produced by different genetic groups and finishing systems. Samples of the *Longissimus thoracis* were collected from 160 commercial bulls of the *B. taurus* (n = 75) and *B. indicus* (n = 85) groups, finished either on pasture (n = 46) or with grain supplementation (n = 114) and analysed by standard procedures. Data were analysed by discriminant analysis using a stepwise procedure, to select the meat characteristics that better contribute to discriminate the various groups. Our results indicate that fatty acid profiles of meat had better discriminating ability than physicochemical properties, especially to identify meat from animals finished on grain or pasture. The overall discrimination of meat from different genetic groups was achieved with a slightly lower reliability. Nonetheless, our results show that reliability of allocation to genetic group can be improved if prior information on finishing system is considered. These results are of high importance because they can be incorporated as tools to assess the authenticity of beef, particularly in meat certification programs.

## 1. Introduction

In addition to price, consumer decisions regarding the choice of a specific meat product are usually based on its assumed quality and healthiness [1], and these are often related with the genetic background and production system from which the product originates. Beef produced under extensive production systems is usually perceived to have a healthier fatty acid profile, and feeding strategies based on pasture-finishing have been developed with this goal [2,3]. Various studies on consumer preferences regarding meat and meat products indicate that consumers generally state that they prefer grass-fed beef, which is perceived as healthier and with a lower environmental impact, and also more in line with consumer concerns with animal welfare issues [4,5].

In parts of the world where tropical climate prevails, taurine cattle are often crossed with *B. indicus*. In Brazil, which is one of the leading producers and exporters of beef in the world [6], nearly 80% of the herd is believed to have some degree of *B. indicus* influence [7]. A decline in meat tenderness in zebu crosses has been well demonstrated [8,9,10], but differences in other physicochemical properties and fatty acid (FA) profiles of meat produced by *taurus* and *indicus* cattle depend on the feeding system used [11,12]. The fact is that there is an image, either real or perceived, that meat produced by *B. indicus* cattle tends to be of lower quality than meat from taurine breeds, and this has led some labelling systems to exclude zebu meat from their certified brands, thus discouraging the sale of such meats in top-quality markets [13].

In spite of the significance of overall mean differences between quality traits of meats originating from different genetic groups or finishing systems, the variability among individual meat samples from the same group tends to be large, which may hinder the ability to reliably distinguish meat samples according to their genetic origin or production system. However, such distinction is essential if certification programs are adopted and appropriately verified [14], and various tools such as physical characteristics, chemical markers, genetic polymorphisms, etc., have been proposed for sample verification and assignment [15].

Canonical discriminant analysis is designed to allow the allocation of observations to their group of origin, which is achieved by the use of classification functions, derived to maximize the distance between the groups analysed [16]. In these analyses, the possibility of an observation being misclassified into a different group is expected to be minimum, as the axes are computed according to their ability to maximize class-separation. 

Several studies have attempted to use multivariate approaches to assess the various factors contributing to variability in meat quality due to genetic background [17,18,19,20] and feeding system [21,22,23,24]. Nevertheless, to our knowledge no comprehensive analysis has compared meat quality traits in *taurus* and *indicus* cattle finished on pasture or grain, with the goal of assessing the suitability of physicochemical properties and lipid profiles of meat as tools to be used to discriminate between meats of different origin. On the other hand, in large genetic groups such as *B. indicus* or *B. taurus*, it is likely that differences in meat quality between breeds of the same genetic group may be important [25,26], which could generate some background noise and cause discrimination between samples from different genetic groups to be more difficult. Therefore, if the comparisons are intended to occur between the broad genetic groups, it is sensible to combine information derived from different breeds of the same group in the comparisons built for discrimination, to enlarge the scope of the discriminant functions.

The objective of this study was to assess the usefulness and reliability of physicochemical properties and fatty acid profiles as sources of information to discriminate between meat produced by commercial *taurus* or *indicus* cattle, finished on pasture or grain. 

## 2. Materials and Methods 

### 2.1. Animals and Management

An experiment was carried out in the State of Minas Gerais, Brazil, to assess various factors related to meat quality traits in commercial beef cattle. Details on the experimental procedures followed in this study have been previously described in detail by our group [11,12] and are briefly summarized here. A group of commercial bulls (n = 160) was slaughtered when they reached an age between 26 and 40 months, and their carcass weight ranged from 270 to 300 kg. The bulls were sampled to represent the commercial animals typically slaughtered in Brazil, and included animals of the genetic groups *B. taurus* (n = 75) and *B. indicus* (n = 85), which had been finished on pasture (n = 46, of which 20 were *B. taurus* and 26 *B. indicus*), or on grain (n = 114, of which 55 were *B. taurus* and 59 *B. indicus*). The sampled bulls were selected to represent some of the major cattle breeds raised in Brazil, such that the Angus, Holstein and Simmental breeds were included in the *B. taurus* sample and Nelore, Gir and Tabapuã cattle were included to represent *B. indicus.*

Animals finished on pasture were raised on grass (*Brachiaria brizantha* cv. Marandu, *Brachiaria decumbens*, *Brachiaria humidicula* and *Panicum maximum* Jacq.) in the Brazilian state of Minas Gerais. Those finished with grain had a finishing period of about 90 d where supplementation with 50% roughage (bagasse sugar cane) and 50% concentrate (consisting of 40.5% corn, 6% soybean meal, 1.5% urea and 2% minerals and vitamins) was available ad libitum. 

### 2.2. Experimental Procedures 

During the growth and finishing period, as well as at slaughter, animals were treated and handled humanely, following the guidelines of State of Sao Paulo (Brazil) law number 11977/2005. Animals were slaughtered in a meat packing plant, certified for beef export, and samples of about 500 g were collected at 24 h post mortem from the M. *longissimus thoracis* between the 5th and the 7th rib of the carcass. The individual samples were vacuum-packaged and frozen at −20 °C for further analyses. After about 30 d of storage, samples were thawed at 4 °C for 24 h, and were either immediately analysed or submitted to ageing for 10 days at 1 °C. 

Steaks with a thickness of 2.5 cm were obtained from both fresh and aged samples, by perpendicular sectioning of the *longissimus thoracis*. After blooming, colour coordinates were obtained with a Minolta Cr-400 colorimeter (Minolta Camera Co., Ltd., Osaka, Japan), and the CIE Lab colour scores indicating lightness (L*), redness (a*) and yellowness (b*) were obtained in both fresh (24 h post mortem) and aged (10 days post mortem) meat samples. Steaks were grilled until reaching an internal temperature of 65 °C, and the difference in weight before and after grilling was considered to correspond to cooking loss. Peak Warner-Bratzler shear force was measured in grilled meat, using a texturometer TA-XT2 (Stable Micro System, Surrey, England).

Samples of fresh meat were minced for chemical analyses. Moisture, protein, fat, ash and cholesterol were determined by standard procedures, as described by [11]. The total lipids extracted from meat samples were used to prepare FA methyl esters, as detailed by [12]. The FA methyl esters were submitted to GLC on an Agilent HP 6890 chromatographer (Agilent Technologies Inc., Palo Alto, CA, USA) equipped with a flame ionization detector and a 100 m capillary column, following the methods described by [27]. The chromatographic resolution of the *trans* 18:1 isomers was not complete, such that the *trans*-6, *trans*-7 and *trans*-8 18:1 isomers coeluted in one peak (hereafter named 18:1 *trans*-6,-7,-8), and the resolution of the *trans*-10, *trans*-11 and *trans*-12 was not complete in many samples (which were thus summed and are hereafter named 18:1 *trans*-10,-11,-12). The 17:1 *cis*-9 also co-eluted the 18:1 dimethylacetal. The individual FA were expressed as a percentage of total identified FA and afterwards grouped as saturated FA (SFA), monounsaturated FA (MUFA) and polyunsaturated FA (PUFA).

For the purposes of discriminant analyses, some of the FA were analysed as groups, as follows:

n-3 group = 18:3n-3 + 20:5n-3 + 22:5n-3 + 22:6n-3

n-6 group = 18:2n-6 + 20:3n-6 + 20:4n-6 + 22:2n-6

Biohydrogenation group = 18:1 *trans*-6,-7,-8 + 18:1 *trans*-10,-11,-12 + 18:1 *cis*-12 + 18:1 *cis*-13 + 18:1 *cis*-14 + 18:2 *cis*-9, *trans*-11 + 18:2 *trans*-11, *cis*-15

Microbial FA = iso14:0 + iso15:0 + anteiso15:0 + 15:0 + iso16:0 + iso17:0 + anteiso17:0

### 2.3. Statistical Analyses

The IBM SPSS Statistics for Windows, Version 26.0 (IBM Corp., Armonk, NY, USA) package was used for statistical analyses. A series of canonical discriminant analyses were performed in order to develop functions that maximize the ability to distinguish between meat originating from the two genetic groups and two finishing systems analysed, as well as from the combination of genetic group and finishing system. These analyses were carried out considering as discriminant factors either the physicochemical properties or the FA profiles of meat, as well as their joint influence. The discriminant analyses were implemented with a stepwise procedure, using *p* < 0.05 for a variable to enter the function and *p* > 0.10 to be removed. The estimated Wilk’s lambda of each model was used as an indicator of the proportion of variability not explained by the discriminant functions. The reliability of the discriminant analyses was also assessed by the percentage of observations correctly allocated to their group of origin when a discriminant function is used. This was achieved both by direct application of each function or set of functions to the full data set, and by implementing crossvalidation with a jacknife procedure, where each observation is sequentially removed from the data set and is then allocated to a group based on the discriminant function developed when this observation was excluded.

## 3. Results

The descriptive statistics for meat physiochemical properties and fatty acid profiles in the 160 samples used in our study are in Table 1, pooled across genetic groups and finishing systems. The mean intramuscular fat content of meat was nearly 6.4% and the more abundant FA were 16:0, 18:0 and 18:1c-9. The mean shear force was about 8 kg at 24 h and declined to nearly 5.6 kg after 10 d of ageing, while cooking loss remained nearly the same at 24 h and 10 d post mortem. As would be expected in meat samples collected from heterogeneous commercial animals, the coefficient of variation (CV) was considerable for most physicochemical variables, especially for fat content, meat yellowness and shear force. For fatty acid profiles, the CV tended to be lower for the more abundant fatty acids, with the exception of 18:0 where the CV was about 18%. More detailed results, as well as the assessment of the influence of genetic group and finishing system on meat physicochemical properties and fatty acid profiles can be found in [11,12]. Here, our focus was to evaluate the ability of correctly allocating a meat sample to its genetic group and finishing system, based on its characteristics.

**Figure 1:**
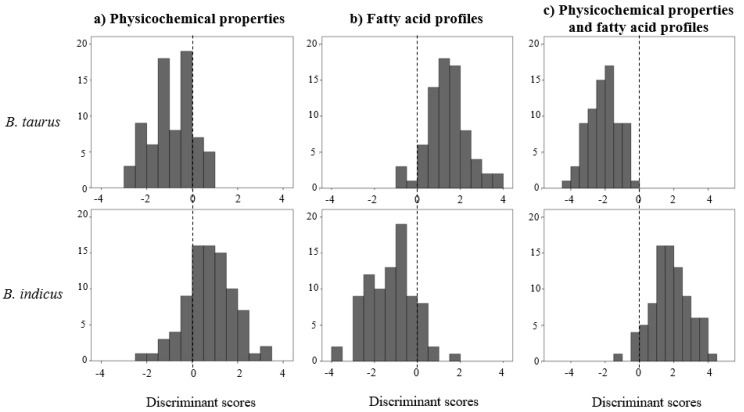
Distribution of discriminant scores in *l. thoracis* samples from *B. taurus* and *B. indicus* bulls based on: (**a**) meat physicochemical properties; (**b**) fatty acid profiles; (**c**) physicochemical properties and fatty acid profiles.

In our study, the stepwise discriminant analyses converged for all traits and discrimination criteria evaluated. In the analyses, to discriminate between genetic groups based on physicochemical properties, model fitness was not very good, as implied by a large Wilk’s lambda of 0.582 (Table 2). The relative weight of the variables in the discriminant function (Table 3), represented by both the unadjusted and the standardized coefficients, indicates that cholesterol is the major discriminant factor of genetic groups, followed by shear-force of meat at 24 h and yellowness after 10 days of ageing. The application of the discriminant function to genetic groups considering physicochemical data for the meat samples analysed resulted in the distribution of discriminant scores shown in Figure 1 for *B. indicus* and *B. taurus* samples. It is clear that discrimination of genetic groups based on physicochemical properties of meat is very inaccurate, with large overlapping of the two distributions.

When genetic groups were discriminated based on their FA profile, the fitness of the model improved but it was still modest (Wilk’s lambda of 0.363, Table 2), and the standardized coefficients (Table 3) indicate that the variables with higher discriminant power are n-3, 16:1 *cis*-7, 17:1 *cis*-9, intermediaries of the biohydrogenation pathways and 14:0. Relative to physicochemical properties of meat, the ability to discriminate between genetic groups improved substantially when FA profiles were used, as indicated by the better separation of the distribution of discriminant scores for each of the genetic groups (Figure 1). Still, some overlapping of the distributions could be observed, implying that a poor discrimination ability of genetic groups was achieved based on FA profiles.

When physicochemical properties of meat were combined with FA profiles to discriminate between samples from *B. indicus* and *B. taurus*, the suitability of the discriminant function improved when compared with the results obtained with each group of traits individually, but the estimated Wilk’s lambda was still high (0.208, Table 2), indicating that reliability of the discrimination procedure remained low. Of the 14 variables included in the discriminant function (Table 3), the ones with higher discriminating power for genetic groups were the amount of FA of microbial origin, n-3 FA, 18:0, 17:0 and cholesterol. The weights to be used in the discriminant function to distinguish between *B. taurus* and *B. indicus* meat are in Table 3, and their application to the experimental data resulted in the distributions of discriminant scores shown in Figure 1, which indicates a reasonable separation between the two genetic groups, with a slight degree of overlapping.

**Figure 2:**
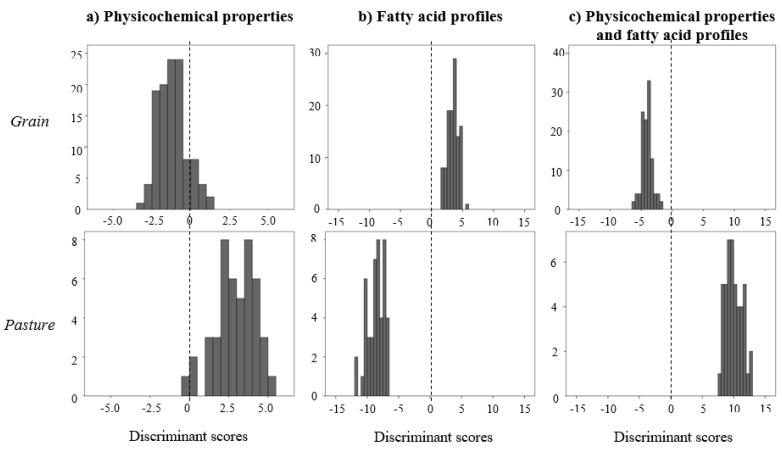
Distribution of discriminant scores in *l. thoracis* samples from bulls finished on grain or pasture, based on: (**a**) meat physicochemical properties; (**b**) fatty acid profiles; (**c**) physicochemical properties and fatty acid profiles.

Discrimination among finishing systems based on physicochemical properties was more reliable than the discrimination of genetic groups, with a smaller Wilk’s lambda (0.222, Table 2). The discriminant function in this case was largely determined by protein content of meat, but also included cholesterol and meat redness, yellowness and shear force at 24 hours (Table 4). Applying the discriminant function resulted in the distribution of discriminant scores for the samples analysed shown in Figure 2. Even though the two groups differ when discrimination is based on physicochemical properties alone, the two distributions still have some overlapping, indicating that the distinction of the two groups cannot be made reliably based on these criteria. When discriminant analysis of finishing systems was based on fatty acid profiles, the situation improved enormously, with a dramatic decline in Wilk’s lambda (λ = 0.032, Table 2). The factors with stronger discriminant ability in this case, as indicated by the standardized coefficients (Table 4), were the concentration of 16:1 *cis*-9, followed by FA of microbial origin, n-3, n-6, 17:1 *cis*-9, 12:0 and 18:0. Using the estimated discriminant function resulted in a complete separation of the groups of grain- and pasture-finished animals, ensuring a high accuracy in allocation to group of origin (Figure 2). Combining in the discriminant function the physicochemical properties and FA profiles of meat improved slightly the ability to distinguish the two finishing systems relative to the use of FA profiles alone, with a minor decline in Wilk’s lambda and similar degree of separation of the two groups in the distribution of discriminant scores (Figure 2). The variables included in the discriminant function in this case (Table 4) were essentially the same already included when only FA profiles were considered, plus a few additional FAs (particularly 16:0 and 18:2 *cis*-9,*t*-11) and meat fat and moisture.

**Figure 3:**
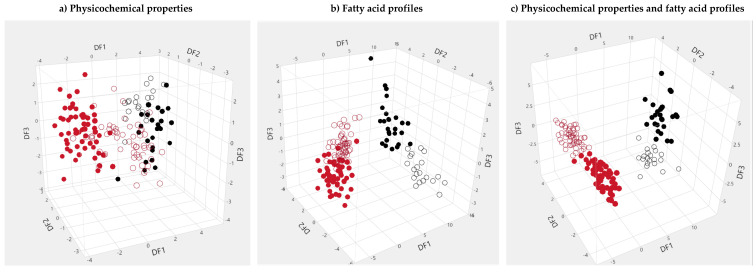
Distribution of discriminant scores attributed according to three discriminant functions (DF1, DF2 and DF3, respectively) applied to 160 *l. thoracis* samples from *B. indicus* and *B. taurus* bulls finished on grain or pasture, based on: (**a**) meat physicochemical properties; (**b**) fatty acid profiles; (**c**) physicochemical properties and fatty acid profiles. **◯**—*Taurus*, pasture; **◯**—*Taurus*, grain; ●—*Indicus*, pasture; ●—*Indicus*, grain.

The possibility of distinguishing between meats resulting from combinations of genetic group × finishing system was investigated, and since there were four combinations, three discriminant functions were estimated. Meat physicochemical properties provided a model with modest fitness, with Wilk’s lambda of 0.07 (Table 2), and the factors with the largest influence in the discriminant functions were protein, cholesterol and meat yellowness at 10 d of ageing (Table 5). The use of the discriminant functions estimated from physicochemical properties of meat to compute a discriminant score per animal resulted in some grouping of animals according to their genetic group × finishing system combination (Figure 3), but a large degree of admixture of the various groups was still observed. Therefore, assignment to genetic group × finishing system combinations based on physicochemical properties of meat was not a reliable possibility, given the high degree of overlapping of the various groups.

The use of fatty acid profiles provided a much better discrimination of genetic group×finishing system combinations, with a model showing good fitness, expressed in a Wilk’s lambda of 0.004 (Table 2). In this case, the variables with stronger influence in discriminant function 1 (Table 5) were 16:1 *cis*-9, 14:0, 18:2 *cis*-9, *t*-11, intermediate FA in the biohydrogenation pathways, 12:0 and n-3 FA. In discriminant function 2 the major factors were n-3, 17:0, 17:1 *cis*-9 and microbial FA, while for discriminant function 3 the major FAs were n-3, 17:1 *cis*-9, 16:1 *cis*-9 and 17:0. The application of the discriminant functions based on the estimated coefficients reported in Table 4 for FA profiles resulted in the dispersion of discriminant scores shown in Figure 3. The separation of groups according to finishing system is very clear and essentially a result of discriminant function 1. On the other hand, some separation according to genetic group within finishing system is also visible, even though not as clear as for finishing system, and results essentially from discriminant function 2. In grain-finished animals, discriminant function 3 provided a good separation of the two genetic groups.

The combination of meat physicochemical properties and FA profiles in discriminant analysis was the best approach to assess the differentiation among genetic group × finishing system combinations. The model fitness was excellent (Wilk’s lambda of 0.002, Table 2) and the factors with a stronger weight in function 1 were essentially those described for the first discriminant function when only FA were considered in the analysis (Table 5). Discriminant function 2 was most influenced by cholesterol content, 16:1 *cis*-9 and 14:0, while the variables with more influence in function 3 were n-3, 17:0, 17:1 *cis*-9 and microbial FA. The use of the estimated discriminant functions resulted in a very good separation of the four groups analysed (Figure 3), such that discriminant function 1 separated groups according to finishing system, function 2 separated *taurus* from *indicus* in grain-finishing and function 3 separated the two genetic groups in pasture-finishing.

**Figure 4:**
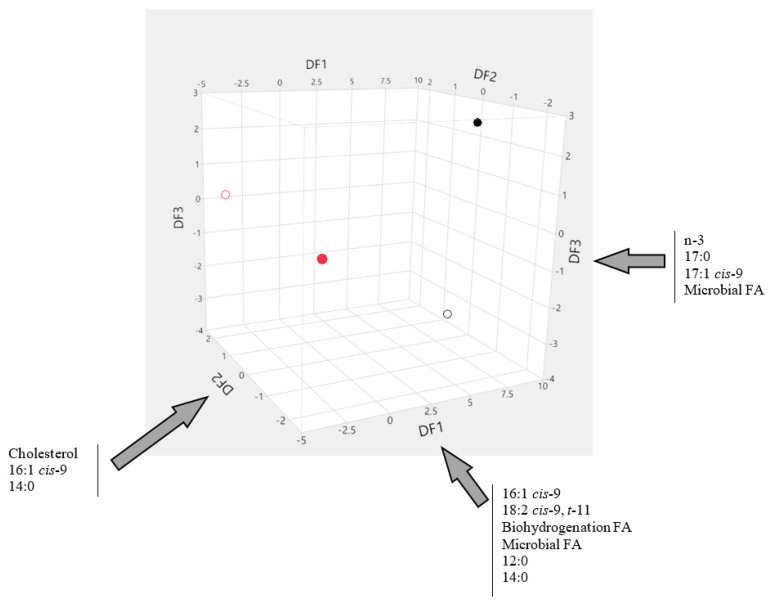
Graphical representation of the discriminant scores at the centroid of each genetic group × finishing system cluster according to three discriminant functions (DF1, DF2 and DF3, respectively) constructed based on physicochemical properties and fatty acid profiles, with indication of the most important variables involved in each discriminant function. **◯**—*Taurus*, pasture; **◯**—*Taurus*, grain; ●—*Indicus*, pasture; ●—*Indicus*, grain.

The separation of the various groups by discriminant functions can be further assessed by ascertaining how the centroid of each genetic group × finishing system cluster is positioned according to the three discriminant functions (DF1, DF2 and DF3), constructed based on physicochemical properties and fatty acid profiles, as represented in Figure 4. Additionally represented in this Figure are the most important variables involved in each discriminant function, confirming the importance of FA as discrimination criteria, particularly the cis-9 group and microbial FA. On the other hand, the only physicochemical characteristic with importance in discrimination was cholesterol, which allowed the separation between *taurus* and *indicus* under grain-finishing. 

The various models differed considerably in their ability to correctly assign an individual to its group of origin (Table 6). The poorest results were consistently obtained with discrimination based on physicochemical properties of meat, in particular if the assignment to genetic group was intended. In this scenario, nearly 80% of the samples were assigned to their genetic group when crossvalidation was used. The reliability of correct allocation based on physicochemical properties improved in the assignment to finishing system, where the percentage of correct allocations was about 95%. Using FA profiles improved the ability to correctly allocate to the group of origin, which increased to about 90% for genetic groups and 100% for finishing systems. The combination of physicochemical properties and FA profiles improved the reliability of allocation to genetic group to about 95%, while for finishing system the percentage of correct allocations was maintained at 100%.

The allocation of samples to the correct genetic group×finishing system of origin was unreliable if only physicochemical properties were used, but achieved a very high accuracy when the discrimination was based on a function derived from physicochemical properties and FA profiles of meat. Based on the combination of traits, the ability to allocate samples to the correct group of origin ranged from 96 to 100% (Table 6). Among the 160 animals sampled, the few misclassifications detected resulted from animals that were classified in the wrong genetic group but in the right finishing system (results not shown). These results confirm the very clear discrimination between meat resulting from either one of the finishing systems, but a slight degree of overlapping was observed between the two genetic groups.

## 4. Discussion

It has been well documented that physicochemical properties and fatty acid profiles of beef are influenced by both genetic group and finishing system, which could allow the discrimination of meat samples according to their origin and thus provide tools for meat certification. For example, meat from *B. taurus* is generally tenderer than meat produced by *B. indicus* [9,10] and pasture finishing leads to higher amounts of PUFA [28,29]. However, it has been argued that the within-breed variability for meat quality traits is often more important than the difference among breed means [8], which would hamper the ability to assign individual samples to their correct breed with reasonable accuracy, and the same could be true for finishing system. One alternative would be the use of discriminant analysis, allowing the prediction of group membership from a set of continuous predictors, and this approach has been used with a variable degree of success to distinguish between meats from different cattle breeds [18,19,20,26,30] and different finishing systems in various ruminant species [3,21,22,23,24]. Nevertheless, to our knowledge the ability to discriminate between taurine and indicine meat and among meats originating from combinations of different genetic groups and finishing systems has not been investigated so far.

In our analyses, the discrimination among finishing systems was achieved with very high accuracy when FA profiles were used to build the discriminant functions, with higher discriminating ability for 16:1 *cis*-9, microbial FA, 17:1 *cis*-9, 18:0, n-3 and n-6 FA. However, discrimination among finishing systems based on physicochemical properties was not so reliable, and was prone to some misclassifications. Combining FA profiles and physicochemical properties in the discriminant function did not improve the discriminating ability between finishing systems relative to the use of FA profiles alone, as these were already very reliable. These results were anticipated, as finishing system is expected to have a much stronger influence on FA profiles of meat than on its physicochemical properties [2,11,12,31]. The results of the present study provide strong evidence that meat properties, in particular fatty acid profiles, can be used as tools to accurately identify the finishing system from where a meat sample originated, and are thus extremely useful as validation tools in meat certification programs.

On the other hand, the ability to discriminate meats according to genetic group was not as reliable as it was for finishing system, and it was only feasible with good accuracy when FA profiles and physicochemical properties were combined in the discriminant function, where more than 95% of the samples could be correctly allocated to genetic group. The factors with higher discriminating ability in this case were microbial FA, n-3 FA, 17:0, 18:0 and cholesterol, largely reflecting the differences among *taurus* and *indicus* cattle in rumen biohydrogenation [12,32]. These results further indicate that, although tenderness is generally assumed to be, on average, less desirable in *B. indicus* meat [8,9], the variability observed among meat samples from different animals is quite large, such that the ability to discriminate meats according to their genetic group of origin is not feasible based on physicochemical properties alone. Our results point towards the need to possibly reconsider some of the prejudice that has prevailed towards zebu meat [13], which does not seem to be sufficiently justified based on experimental data.

When the goal was to discriminate between combinations of genetic group × finishing system, the use of meat physicochemical properties alone was not effective to obtain an accurate distinction between groups, but an improvement in discrimination ability was achieved when FA profiles were used instead, even though some misclassifications were still observed, especially in grain-finished animals. With the combination of FA profiles and meat physicochemical properties, the discrimination among the four genetic group × finishing system combinations was very accurate, with only a few *indicus* animals misclassified. The very high accuracy (>95% correct classifications) achieved under these circumstances indicates that meat samples can be assigned reliably to the genetic group × finishing system combinations considered here. The first discriminant function allowed a complete separation of samples originating from each of the two finishing systems, and included as main discriminating factors the *cis*-9 FA, as well as the FA resulting from biohydrogenation and of microbial origin, 12:0 and 14:0. The second discriminant function allowed the separation of samples from *taurus* or *indicus* bulls, and included as main factors cholesterol, 14:0 and 16:1 *cis*-9. This function was unambiguous in discriminating among *taurus* and *indicus* samples in grain-finishing, but some overlapping remained for the two genetic groups under pasture-finishing. However, this issue was disentangled by the third discriminant function, which specifically separated the two genetic groups under pasture-finishing, and included as main discriminating factors the n-3, 17:0, 17:1 *cis*-9 and microbial FA. Thus, the distinction of meat originating from the four genetic group × finishing system combinations could be achieved by discriminant analysis with high reliability.

These results are useful to develop and apply discrimination tools that can be used in certification programs, to ensure meat authenticity regarding genetic background and feeding system throughout the production chain. Along with other traceability mechanisms, discriminant analyses should be a key component of a robust follow-up of the meat supply-chain, enabling the detection of fraudulent situations, thus adding value to quality labelling programs.

Further studies are needed to clarify how different finishing alternatives, in terms of diet composition and length of the finishing period [3,25,33,34], may affect the patterns identified here and how other chemical markers such as terpenes and carotenoids [35], as well as stable isotope ratios [14,36], may be used in discriminating finishing systems. On the other hand, the possible discrimination among meat from *indicus* or *taurus* cattle may be further enhanced by combining meat properties with the information provided by genetic markers that may enable breed assignment [37,38,39]. Still, the discrimination of meat from crossbred *taurus × indicus* animals represents an additional challenge, as it has been shown that heterosis plays a role in various meat characteristics, which may differ across finishing systems [40]. Thus, the discrimination of meat samples obtained from purebred as well as crossbred *taurus × indicus* cattle should be investigated in future studies, possibly in combination with genetic markers, as these should allow a better quantification of the diverse contributions from *taurus* and *indicus* in crossbred individuals, thus accounting for various degrees of genetic inflow from each of the genetic groups.

## 5. Conclusions

The results of discriminant analyses with meat samples from *B. indicus* and *B. Taurus* finished on pasture or grain indicate that meats originating from each of the finishing systems can be reliably discriminated, essentially on the basis of their fatty acid profile. The global distinction of meat from the two genetic groups is not as reliable, but discrimination can be enhanced by prior knowledge of the finishing system used. In this case, our results show that it is feasible to distinguish with high reliability between meat samples from *B. indicus* and *B. taurus* finished on pasture or grain, with fatty acid profiles being better discriminating factors than physicochemical properties. These results are of high importance because they can be incorporated as tools to assess the authenticity of beef, especially in meat certification programs. Undoubtedly, in combination with other traceability mechanisms, discriminant analysis is a powerful tool for a robust follow-up of the meat supply-chain, enabling the detection of fraudulent situations, thus adding value to quality labelling programs.

## Figures and Tables

**Table 1:** Mean and coefficient of variation (CV) for meat chemical composition, colour parameters, physical properties and fatty acid profiles (in % of total fatty acids) in *l. thoracis* samples obtained from 160 *B. indicus* and *B. taurus* bulls finished on grain or pasture.

Group	Variable	Mean	CV (%)
Meat chemical composition	Moisture (%)	72.63	2.8
	Protein (%)	19.08	8.9
	Ash (%)	0.90	20.0
	Fat (%)	6.39	49.0
	Cholesterol (mg/100 g)	49.27	32.5
Meat colour parameters	L* 24 h	32.76	7.4
	a* 24 h	20.00	7.8
	b* 24 h	4.23	27.4
	L* 10 d	33.61	7.9
	a* 10 d	16.51	12.6
	b* 10 d	4.87	28.5
Meat physical properties	Cooking loss 24 h (%)	29.06	16.0
	Shear Force 24 h (kg)	7.98	21.2
	Cooking loss 10 d (%)	30.20	17.1
	Shear Force 10 d (kg)	5.64	27.0
	pH 24 h	5.85	3.1
Fatty acids (FA, %)	Microbial FA	2.26	21.2
	Biohydrogenation FA	4.06	23.2
	n-3 FA	1.22	93.4
	n-6 FA	3.49	40.4
	12:0	0.07	42.9
	14:0	3.04	21.1
	14:1c-9	0.49	36.7
	16:0	24.62	8.0
	16:2	0.34	26.5
	16:1c-9	2.79	23.3
	17:0	1.21	13.2
	17:1c-9	1.03	72.8
	18:0	20.42	17.8
	18:1c-9	32.64	8.9
	18:1c-11	1.20	26.7
	20:0	0.18	27.8
	20:1-c13	0.28	60.7
	18:2-c9,t-11	0.48	27.1

**Table 2:** Estimated Wilk’s lambda in discriminant analyses of meat samples according to genetic group, finishing system or genetic group × finishing system combinations based on meat physicochemical properties, fatty acid profiles or both.

Discriminant Factors	Genetic Groups	Finishing Systems	Genetic Group × Finishing System Combinations
Physicochemical (PC) properties	0.582	0.222	0.070
Fatty acid (FA) profiles	0.363	0.032	0.004
PC properties and FA profiles	0.208	0.024	0.002

**Table 3:** Estimated coefficients of the canonical discriminant function on the predictor variables, in discriminant analyses of genetic groups based on meat physicochemical properties, fatty acid profiles or both.

Discrimination Criteria	Discriminant Function		
	Variable	Unstandardized Coefficients	Standardized Coefficients
Physicochemical properties	Cholesterol	0.057	0.781
	Shear Force 24 h	0.340	0.525
	b* 10 d	−0.312	−0.413
	(Constant)	−3.981	-
Fatty acid profiles	Biohydrogenation FA	−1.195	−0.919
	n-3	−1.382	−1.570
	12:0	20.192	0.659
	14:0	−1.429	−0.892
	16:0	0.322	0.614
	16:1*c*-7	11.810	1.119
	17:0	−3.027	−0.484
	17:1*c*-9	1.260	0.952
	20:1*c*-13	3.545	0.599
	(Constant)	−1.187	-
Physicochemical properties and fatty acid profiles	Microbial FA	−2.589	−1.240
	Biohydrogenation FA	0.586	0.451
	n-3	0.893	1.014
	12:0	−19.301	−0.630
	14:0	0.908	0.567
	14:1*c*-9	2.662	0.473
	17:0	4.638	0.741
	18:0	0.255	0.863
	18:2*c*-9,*t*-11	5.147	0.635
	Moisture	0.156	0.319
	Ash	−1.286	−0.222
	Cholesterol	0.056	0.768
	Shear Force 24 h	0.171	0.265
	b* 10d	−0.244	−0.322
	(Constant)	−26.590	-

**Table 4:** Estimated coefficients of the canonical discriminant function on the predictor variables, in discriminant analyses of finishing systems based on meat physicochemical properties, fatty acid profiles or both.

Discrimination Criteria	Discriminant Function		
	Variable	Unstandardized Coefficients	Standardized Coefficients
Physicochemical properties	Protein	1.074	0.953
	Cholesterol	−0.024	−0.366
	a* 24 h	−0.239	−0.357
	b* 24 h	0.201	0.233
	Shear Force 24 h	0.114	0.189
	(Constant)	−16.266	-
Fatty acid profiles	Microbial FA	−1.977	−0.792
	n-3	−0.983	−0.564
	n-6	0.546	0.656
	12:0	30.608	0.623
	14:0	−0.941	−0.503
	16:1c-7	−6.184	−0.320
	16:1c-9	2.630	1.531
	17:0	2.404	0.386
	17:1c-9	−2.088	−0.683
	18:0	0.195	0.668
	18:1c-11	−2.508	−0.528
	20:0	7.111	0.334
	20:1c-13	4.135	0.432
	(Constant)	−5.044	-
Physicochemical properties and fatty acid profiles	Moisture	0.357	0.691
	Fat	0.435	1.015
	L* 24 h	−0.088	−0.214
	Microbial FA	−1.798	−0.721
	Biohydrogenation FA	0.779	0.695
	n-3	−0.844	−0.484
	n-6	0.535	0.642
	12:0	26.423	0.538
	14:0	−1.020	−0.545
	16:0	−0.210	−0.368
	16:2	−8.253	−0.427
	16:1c-9	2.830	1.648
	17:0	3.196	0.512
	17:1c-9	−2.438	−0.797
	18:1-c11	−2.025	−0.426
	20:0	7.619	0.358
	20:1-c13	4.000	0.418
	18:2-c9,t-11	−7.032	−0.741
	(Constant)	−22.621	-

**Table 5:** Estimated coefficients of the canonical discriminant function on the predictor variables, in discriminant analyses of genetic groups × finishing system combinations based on meat physicochemical properties, fatty acid profiles or both.

Discrimination Criteria	Variable	Unstandardized Coefficients			Standardized Coefficients		
		Function 1	Function 2	Function 3	Function 1	Function 2	Function 3
Physicochemical properties	Protein	1.003	0.445	0.205	0.892	0.396	0.183
	Cholesterol	−0.069	0.075	0.024	−0.665	0.716	0.228
	a* 24 h	−0.124	−0.189	−0.123	−0.187	−0.283	−0.185
	Shear Force 24h	0.028	0.256	−0.278	0.042	0.388	−0.421
	b* 10 d	−0.011	−0.029	0.625	−0.015	−0.037	0.812
	(Constant)	−13.410	−10.294	−3.454	-	-	-
Fatty acid profiles	Microbial FA	1.064	−2.227	−1.376	0.423	−0.885	−0.547
	Biohydrogenation FA	−0.906	0.775	−0.244	−0.636	0.544	−0.171
	n-3	1.132	3.052	1.786	0.602	1.622	0.949
	n-6	−0.481	−0.165	0.127	−0.574	−0.197	0.151
	12:0	−29.838	−10.257	12.169	−0.609	−0.209	0.248
	14:0	1.402	1.132	−1.063	0.721	0.582	−0.547
	16:0	0.161	−0.125	0.349	0.271	−0.210	0.589
	16:2	9.052	−1.963	11.573	0.465	−0.101	0.595
	16:1c-9	−2.851	0.818	1.860	−1.341	0.384	0.874
	17:0	−1.901	7.194	4.292	−0.300	1.137	0.678
	17:1c-9	1.839	−3.413	−2.751	0.595	−1.105	−0.891
	18:1-c11	2.185	−0.353	0.896	0.409	−0.066	0.168
	20:1-c13	−3.571	−1.981	0.632	−0.375	−0.208	0.066
	18:2-c9,t-11	7.404	3.278	−0.731	0.715	0.316	−0.071
	(Constant)	−4.327	−8.274	−17.221			
Physicochemical properties and fatty acid profiles	Microbial FA	1.615	0.009	−2.469	0.642	0.004	−0.981
	Biohydrogenation FA	−0.928	−0.078	0.791	−0.651	−0.054	0.555
	n-3	0.572	−0.016	3.268	0.304	−0.008	1.737
	12:0	−31.774	24.073	−2.326	−0.649	0.491	−0.047
	14:0	1.065	−1.172	0.438	0.548	−0.603	0.225
	16:0	0.232	0.267	0.015	0.391	0.450	0.025
	16:2	9.532	8.389	1.755	0.490	0.431	0.090
	16:1c-9	−2.599	1.492	1.722	−1.222	0.702	0.810
	17:0	−1.841	−0.122	8.369	−0.291	−0.019	1.323
	17:1c-9	1.638	−0.419	−4.283	0.530	−0.136	−1.386
	20:0	−8.541	0.954	0.464	−0.381	0.043	0.021
	20:1-c13	−4.655	0.668	−2.159	−0.489	0.070	−0.227
	18:2-c9,t-11	8.447	−3.425	2.122	0.815	−0.331	0.205
	Protein	0.381	0.289	0.078	0.339	0.257	0.069
	Cholesterol	−0.007	−0.086	−0.023	−0.068	−0.826	−0.222
	Shear Force 24 h	0.047	−0.165	0.180	0.071	−0.251	0.272
	(Constant)	−11.095	−9.634	−16.564			

**Table 6:** Percentage of correct allocations to genetic group, finishing system or combination of genetic group × finishing system, with discriminant functions based on meat physicochemical properties, fatty acid profiles or both. Values in ( ) correspond to crossvalidation results, where each observation is assessed after being removed from the data set to compute a discriminant function.

Source of Information	Genetic Group		Finishing System		Genetic Group × Finishing System			
	*Taurus*	*Indicus*	Grain	Pasture	Indicus Pasture	Indicus Grain	Taurus Pasture	Taurus Grain
Physicochemical (PC) properties	81.3 (80.0)	81.2 (78.8)	98.2 (98.2)	93.5 (93.5)	69.2 (69.2)	94.9 (94.9)	75.0 (65.0)	80.0 (80.0)
Fatty acid (FA) profiles	93.3 (92.0)	89.4 (87.1)	100.0 (100.0)	100.0 (100.0)	100.0 (96.2)	93.2 (88.1)	100.0 (100.0)	92.7 (90.9)
PC and FA profiles	100.0 (97.3)	95.3 (94.1)	100.0 (100.0)	100.0 (100.0)	100.0 (96.2)	100.0 (96.6)	100.0 (100.0)	98.2 (98.2)
